# The Site Frequency/Dosage Spectrum of Autopolyploid Populations

**DOI:** 10.3389/fgene.2018.00480

**Published:** 2018-10-23

**Authors:** Luca Ferretti, Paolo Ribeca, Sebastian E. Ramos-Onsins

**Affiliations:** ^1^The Pirbright Institute, Woking, United Kingdom; ^2^Centre for Research in Agricultural Genomics, Barcelona, Spain

**Keywords:** autopolyploidy, dosage distribution, Hardy-Weinberg equilibrium, high-throughput sequencing, site frequency spectrum, heterozygosity, neutrality tests, allelic dosage

## Abstract

The Site Frequency Spectrum (SFS) and the heterozygosity of allelic variants are among the most important summary statistics for population genetic analysis of diploid organisms. We discuss the generalization of these statistics to populations of autopolyploid organisms in terms of the joint Site Frequency/Dosage Spectrum and its expected value for autopolyploid populations that follow the standard neutral model. Based on these results, we present estimators of nucleotide variability from High-Throughput Sequencing (HTS) data of autopolyploids and discuss potential issues related to sequencing errors and variant calling. We use these estimators to generalize Tajima's *D* and other SFS-based neutrality tests to HTS data from autopolyploid organisms. Finally, we discuss how these approaches fail when the number of individuals is small. In fact, in autopolyploids there are many possible deviations from the Hardy–Weinberg equilibrium, each reflected in a different shape of the individual dosage distribution. The SFS from small samples is often dominated by the shape of these deviations of the dosage distribution from its Hardy–Weinberg expectations.

## 1. Introduction

The study of nucleotide variability in polyploid species is a convoluted task that requires solving a number of methodological and analytical difficulties related to the specific nature of the species (detailed in the reviews of Dufresne et al., 2014; Meirmans et al., 2018). The impact of diploidy on the evolutionary dynamics is well-known, but the complexity of the impact of higher ploidy on the genetic variability of polyploid organisms is even higher. An example is provided by autopolyploid species: as they contain copies originating from genome duplication of the same species, the inheritance is expected to be polysomic (all the variants of the same chromosome can pair in the meiosis process) but it is not rare to find preferential pairs (Stift et al., 2008; Chester et al., 2012), resulting in partial polysomic or even disomic inheritance. The different inheritance types, which may simultaneously occur in the same species, could generate differences in the effective population size at different loci and consequently different patterns of genetic variability. Another distinctive aspect of polyploid species that impacts their genetic variability patterns is the process of *double reduction*, where the two copies of the same chromatid migrate to the same gamete (Haldane, 1930). As a consequence, this process will increase drastically the homozygosity of the gametes for the involved segment.

High-Throughput Sequencing (HTS) has facilitated the study of genome data in general and that of polyploid species as well. Still there are difficulties, mainly assigning the sequence reads to homologous (rather than homeologous) loci and/or dealing with relatively high rates of sequencing error (You et al., 2018). The amount of software available in order to correctly assembly and detect variants (e.g., GATK from Broad Institute) is increasing, although the task remains challenging (Mielczarek and Szyda, 2016; You et al., 2018). These methodological problems are expected to be (at least partially) solved in the next years with the technological progress of the sector, including long reads and linked reads to improve phasing and increased throughput of sequencing runs (Dufresne et al., 2014; Shendure et al., 2017).

The study of polyploid variability from HTS data and the development of statistical methods based on these sequencing methodologies are driving current genetic studies of polyploids (Dufresne et al., 2014; Hardy, 2016) and will continue to have a fundamental impact on the field. Nevertheless, still much work is needed, especially on the topic of allelic dosage, that is, the number of copies of each allele in a heterozygous individual (Blischak et al., 2016). Since the development of HTS, a number of studies developing computational and statistical methods that account for polyploidy have been published. Example are statistics to estimate the levels of variability (Ferretti and Ramos-Onsins, 2015) and heterozygosity (e.g., De Silva et al., 2005; Hardy, 2016) with different approaches to take into account the allelic dosage, or the detection of population structure (e.g., Falush et al., 2003; Gao et al., 2007) and comparative measures of these differences between populations/species/individuals (e.g., Jost, 2008; Meirmans and Hedrick, 2011). Arnold et al. (2012) showed that autotetrasomic inheritance can be modeled using a Kingman's standard coalescent (Kingman, 1982). Their results can be generalized to autopolyploid species of different ploidy and are especially useful as a null model to predict the neutral patterns of genetic diversity in polyploid species. Also additional phenomena specific to polyploids, such as *double reduction*, can be modeled in a way resembling partial self-fertilization (Arnold et al., 2012).

Nevertheless, a major gap in the population genetic analysis of polyploid organisms is the application of methods based on the Site Frequency Spectrum (SFS). Of special interest is the generalization to polyploid organisms of Tajima's *D* (Tajima, 1989), Fay and Wu's *H* (Fay and Wu, 2000) and other neutrality tests based on the SFS (Achaz, 2009; Ferretti et al., 2010, 2012). The SFS and the heterozygosity of allelic variants are among the most important statistics for population genetic analysis of diploid organisms and have been commonly used for describing the genetic variability of genomic data and for inferring the parameters of evolutionary models (e.g., Nielsen, 2000). Indeed, the combination of these two statistics (frequency and heterozygosity) describes completely the genotype of a diploid population for a given genomic position.

In this paper we consider a single population of autopolyploid organisms. Compared to the diploid case, the genotypes of variants in polyploid organisms present a more complex structure resulting from a combination of internal spectra for each individual. We discuss this genotype structure and its decomposition into different statistics, including the SFS and a generalization of the distribution of heterozygosity that we call the Site Dosage Spectrum (SDS).

For samples of large size, we argue that the details of deviations from Hardy–Weinberg equilibrium have a relatively small impact on the SFS. The expected value of the SFS of autopolyploid individuals is derived for a panmictic, neutral population of constant size. We also derive the expected value the most general spectrum for autopolyploids, i.e., the joint Site Frequency-Dosage Spectrum (SFDS), which represents a combination of the SFS and the SDS. We use these results as a null model to build estimators of nucleotide diversity and neutrality tests for HTS data and we discuss the robustness of estimators of genetic variability.

For small samples, violations of Hardy–Weinberg in the dosage distribution have a strong impact on the SFS. We show how autopolyploid populations have the potential to harbor a wide range of deviations from Hardy–Weinberg equilibrium due e.g., to inbreeding, population structure, selection, dominance, modes of inheritance, or combinations of these causes. We discuss the impact of some of these violations on dosage and on SFS-based neutrality tests.

A synopsis of symbols and abbreviations used in both text and formulas can be found in Table 1. It should be noted that to the best of our knowledge most of the equations that follow (all but 2, 3, 7, 11, and 13) are original work presented in this paper for the first time. More details about their derivations can be found in the Appendix.

**Table 1:** List of the main symbols and abbreviations used throughout the text.

**Symbol**	**Meaning**
*p*	Ploidy
*n*	Sample size
θ	Genetic variability, i.e., population-scaled mutation rate
ξ_*j*_	Site Frequency Spectrum (SFS) for frequency *j*/*n*
*d*	Allelic dosage
$I_{d}$	Dosage Distribution (DD) for dosage *d*
$p\left({\left\{I_{d}\right\}}_{d=1\ldots p-1}|j\right)$	Site Dosage Spectrum (SDS) for mutations of frequency *j*/*n*
$\psi_{j,\left\{I_{d}\right\}}$	Site Frequency/Dosage Spectrum (SFDS) for frequency *j*/*n* and DD ${\left\{I_{d}\right\}}_{d=1\ldots p-1}$
*r*_*i*_(**x**)	Read depth of the *i*th individual at position *x* along the genome
*c*_*i*_(**x**)	Derived allele count of the *i*th individual at position *x* along the genome

## 2. SFDS structure in autopolyploids

### 2.1. SFS and heterozygosity in diploids

Individuals are often sampled from a wild population without prior studies of the subpopulation structure or phenotypic differences. In this case, it is usually assumed for population genetic analysis that all individuals are equivalent and that any summary statistic should treat all sequences equally. To our knowledge, all existing statistics for sequences sampled from a single populations at the time of this writing—such as estimators of variability, neutrality tests, estimators based on linkage disequilibrium and haplotype-based statistics—rely implicitly on this assumption.

These statistics can also be classified in terms of the number of sites involved in each individual computation. The frequency of a SNP requires information only on the alleles at a single genomic site, while linkage disequilibrium requires a comparison of alleles at two sites. On the other extreme, haplotype statistics require information on all sites in the sequence.

In this manuscript we will focus on the simplest statistics, i.e., those which can be computed independently for each site (and eventually averaged over all sites in the sequence to obtain summary statistics). We will also consider only biallelic variants (one ancestral and one derived/mutated allele present at each site) in our analysis. Biallelic SNPs represent by far the most common type of variant in eukaryotic genomes, hence this assumption is not particularly restrictive. This is true also for autopolyploid organisms, since it relies on the low mutation rates per base and the corresponding low variability at the population level.

A simple explaination for the prevalence of biallelic variants is the following. Under the usual assumptions for the Kingman coalescent, which describes autopolyploid populations as well (Arnold et al., 2012), SNPs are generated by at least a mutation in a given site along the tree. The tree length in coalescent units is a number of order *O*(1), while the effective mutation rate in coalescent units is represented by the parameter of genetic variability θ = 2*pN*_*e*_μ where *N*_*e*_ is the effective population size, *p* is the ploidy and μ is the mutation rate per base. For most eukaryotic organisms, θ is around 10^−3^ (Lynch, 2005). This estimate is based on diploids, but the order of magnitude would be the same for most autopolyploids. The fraction of sites containing a SNP in a finite sample is the product of θ and tree length, and therefore proportional to θ. However, for a triallelic SNP to occur, two mutations should appear on the tree, hence only a fraction *O*(θ^2^) of sites contains a SNP with three or more alleles, i.e., only a fraction *O*(θ) of the SNPs is triallelic. This argument is valid for autopolyploids, but not for allopolyploids, since it does not take into account the divergence between homeologous chromosomes.

In haploid populations, the only statistic based on information at a single position of nucleotide sequences is the frequency of the mutated/derived allele *f*(*x*) at a given site *x*. In fact, once the frequency in the sample is known, the genotypes of all individuals are known up to permutations of the individual. The summary statistic is the so-called SFS, which is the number of sites with a mutation of (derived) frequency *j*/*n* in a sample of *n* individuals, denoted by ξ_*j*_. For the whole population, the equivalent spectrum is the density of sites in the sequence with a mutation of (derived) frequency between *f* and *f* + *df*, denoted by ξ(*f*).

In diploid populations, however, the frequency of a mutation at a given site *x* is not sufficient to fully determine the genotypes of the *n* individuals in the sample. The reason is that each individual can be homozygous for either the ancestral or the mutated allele or it can be heterozygous, i.e., it is characterized by an internal count of the mutated allele at that site (which can be 0, 1, or 2) and a corresponding internal frequency (0, 1/2, or 1). Taken together, all individuals in the sample carry an “internal spectrum” distributed as $I_{d}\left(x\right)$ with *d* = 0, 1, 2, defined as the count of individuals with internal count *d* for the mutation at position *x*, which is of course normalized as $\overset{2}{\underset{d=0}{\sum }}I_{d}\left(x\right)=n$. This individual spectrum is related to the global frequency of the mutation through its mean count $\overset{2}{\underset{d=0}{\sum }}dI_{d}\left(x\right)=2nf\left(x\right)$.

The diploid genotype at position *x* is fully determined by $I_{d}\left(x\right)$ up to permutations of the individuals. Given that $I_{d}\left(x\right)$ has three components (number of ancestral homozygotes $I_{0}$, of heterozygotes $I_{1}$ and of derived homozygotes $I_{2}$) but one is constrained by the number of individuals and another combination corresponds to the frequency, there is only one independent component left, for instance the number of heterozygotes $I_{1}\left(x\right)$. The information contained in this spectrum is therefore equivalent to the two statistics *f*(*x*) and *h*(*x*), where *h*(*x*) is the *heterozygosity* (the fraction of heterozygous individuals in the sample) defined as $h\left(x\right)=I_{1}\left(x\right)/n$.

Heterozygosity is another very well-known statistic in the population genetics of diploid organisms. If the alleles at site *x* are in Hardy–Weinberg equilibrium (i.e., under random mating and without selection), the expected fraction of heterozygotes is given by the standard formula E[*h*(*x*)] = 2*f*(*x*)(1 − *f*(*x*)), i.e., it corresponds to the pairwise nucleotide diversity in the population at that site. Its distribution for a discrete sample is a binomial with the same mean 2*f*(1 − *f*) in terms of the population frequency.

Deviations from the expectation *h* ≈ 2*f*(1 − *f*) are signatures of violations of some of the assumptions of the Hardy–Weinberg equilibrium. For example, a deficit of heterozygotes *h* < 2*f*(1 − *f*) is expected if there is sub-population structure in the sample, violating the “random mating” assumption.

Note that the most general summary single-site statistic for diploids is neither the SFS nor the heterozygosity, but rather the joint site frequency-heterozygosity spectrum ψ(*f, h*) or its corresponding version $\psi_{j,I_{1}}$ for a finite sample. This joint spectrum is defined as the number of sites with a derived variant at frequency *f* = *j*/2*n* and where a fraction $h=I_{1}/n$ of the individuals are heterozygous.

The neutral expectation for this frequency-heterozygosity spectrum in finite samples can be found from the known theory from the frequency spectrum in haploids (Fu, 1995; Ewens, 2004) combined with simple combinatorial arguments applied to the Hardy–Weinberg equilibrium (Weir, 1996). This combination gives

(1) E[ψj,I1]=θ2I1n!I1!j-I12!(n-j+I12)!j2nj

Note the constraint that $j-I_{1}$ should be a multiple of 2.

**Figure 1:**
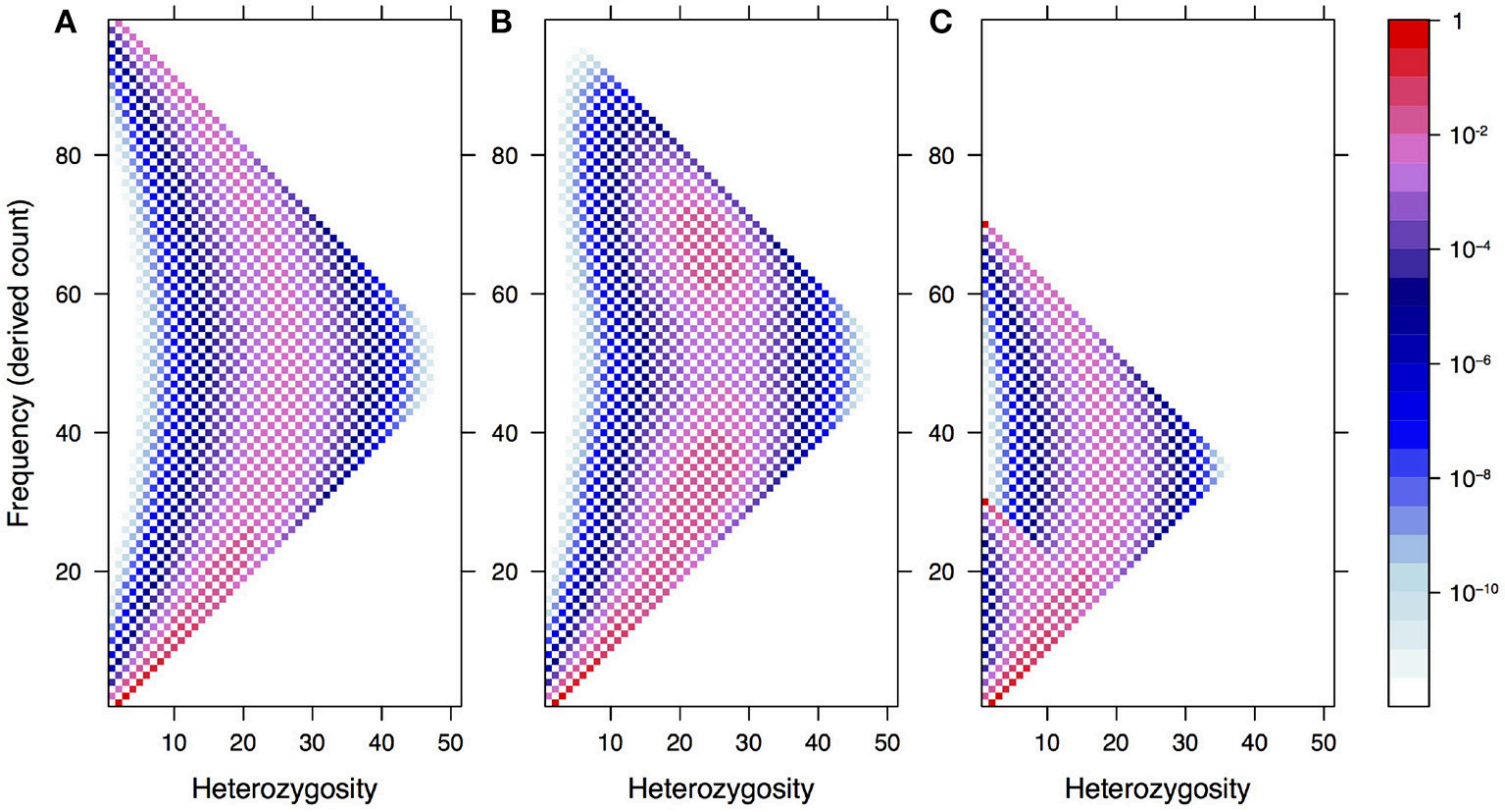
The expected frequency-heterozygosity spectrum for a locus with θ = 1 in a sample of size *n* = 100 from a single population of constant size **(A)** and under two demographic models: recent admixture **(B)** and population structure **(C)**. In both cases, we assume two well-separated populations with divergence equal to θ, the effective population size of the first population being twice the size of the other. In the former case, we assume instantaneous admixture of the two populations and random mating thereafter. In the latter case, the consequence of the absence of mating between different populations is a reduction of heterozygotes in the pooled population, known as the Wahlund effect.

In Figure 1, we illustrate how this spectrum appears under neutrality for a single population of constant size, both in the standard model and under two demographic models: recent admixture and population structure. The latter shows a clear violation of Hardy–Weinberg equilibrium due to a lack of heterozygotes—the so-called Wahlund effect (Rosenberg and Calabrese, 2004).

In diploids, not much attention has been devoted to this joint spectrum, and the two quantities *f* and *h* are usually studied separately. One of the possible reasons is that the Hardy–Weinberg equilibrium is reached in a single generation for diploids, hence heterozygosity and deviations from Hardy–Weinberg equilibrium are affected by phenomena acting on short time scales, while the SFS contains information on evolution at larger scales. However, the difference between these quantities becomes more blurred in autopolyploids, as we will discuss in the rest of this paper.

### 2.2. SFDS in autopolyploids

In autopolyploids, the framework for single-site statistics is reminiscent of the diploid case. The main difference is that at each position of each individual genome the mutated allele can be present in a number of copies from 0 to the ploidy *p*. In polyploids, the frequency of an allele within an individual is often called its *allelic dosage*.

The internal spectrum $I_{d}\left(x\right)$, defined as the count of individuals with allelic dosage *d* for the mutation at position *x*, now covers a broader range of dosages *d* = 0, 1, 2…*p*. For this reason, we will call it the Dosage Distribution (DD). As before, this spectrum is normalized as $\overset{p}{\underset{d=0}{\sum }}I_{d}\left(x\right)=n$ and it is related to the global frequency of the mutation by $\overset{p}{\underset{d=0}{\sum }}dI_{d}\left(x\right)=pnf\left(x\right)$.

Specification of these two conditions can be avoided if we discard the homozygote counts from the DD, since such counts are completely determined by sample size and frequency together with the rest of the DD. The heterozygous part of the SDS plays the same role as heterozygosity in diploids; however, it has the form of a frequency spectrum, hence an additional complexity with respect to the one-dimensional heterozygosity statistic.

**Figure 2:**
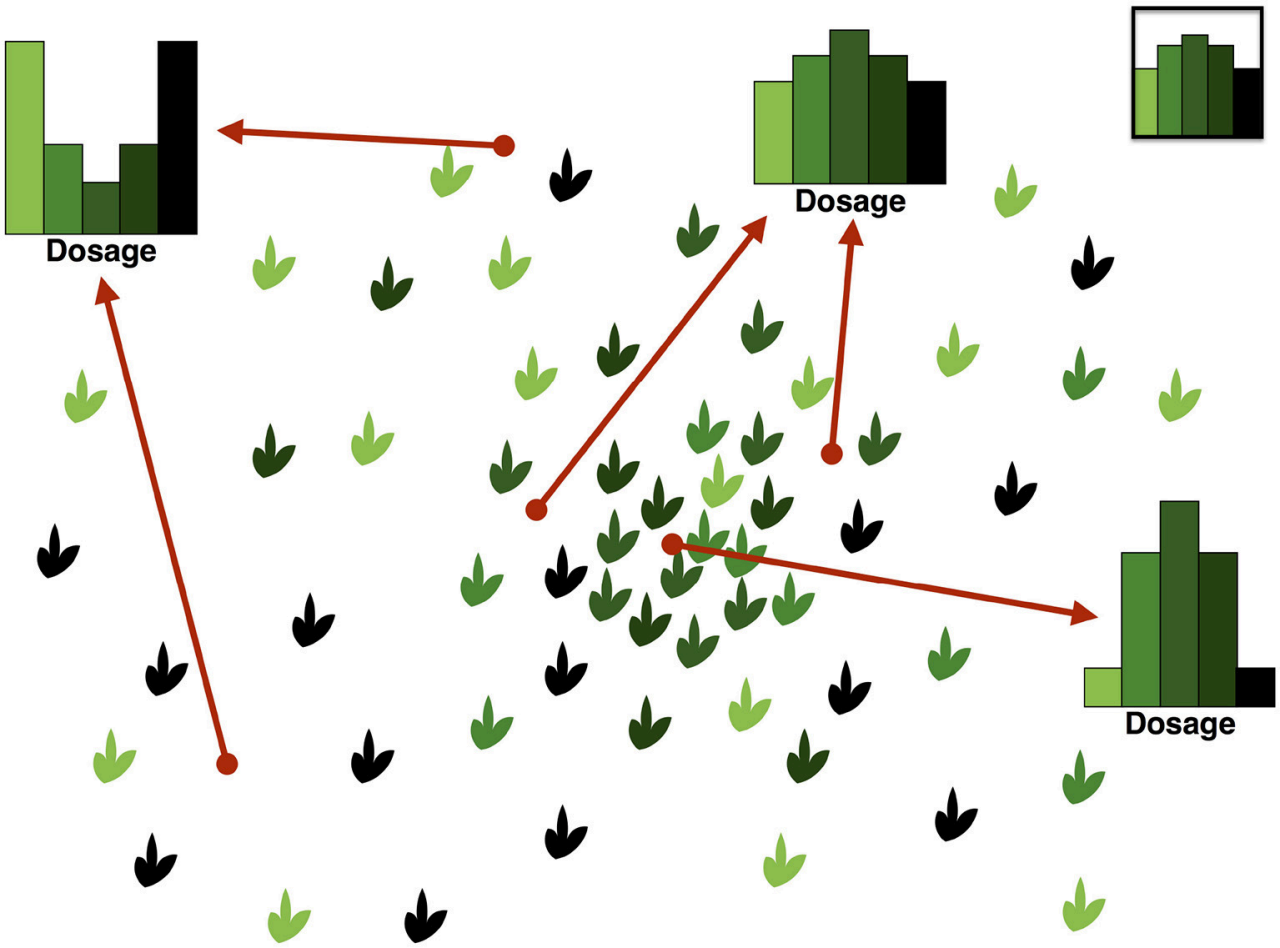
Illustration of the Dosage Distribution (including homozygotes) in a panmictic autotetraploid population with density-dependent selfing rates. In this example, we assume for simplicity that segregating alleles are at intermediate frequency in the population; their dosage in each individual is represented by the color lightness. Since the average frequency is the same everywhere, the average dosage also is. However, by contrast, the DD depends strongly on the sampling location because of variations in the local spatial density. Sampling individuals at random across different locations would result in an average DD like the one in the top-right inset. On the other hand, sampling around a given location would result in different DDs, as illustrated. Locations in the central region tend to have DDs similar to the Hardy–Weinberg ones, while peripheral locations show a large excess of homozygotes because of sampling.

An illustration of the DD and its complexity can be found in Figure 2. In this hypothetical example, we consider a panmictic population with mixed mating (partly selfing, partly outcrossing) and distributed according to a spatial density gradient away from a central region. If the selfing rate depends on the density, being low in dense regions and high in sparse ones, then individuals in dense regions will show a pattern consistent with Hardy–Weinberg equilibrium in the DD, while those in sparse regions will show an excess of homozygotes due to selfing.

For large populations, we can define a normalized DD as $i_{d}=I_{d}/n$. The most general single-site statistic for autopolyploids is therefore the joint Site Frequency-Dosage Spectrum (SFDS) ψ(*f*, {*i*_*d*_}_*d* = 1…*p*−1_) or its discrete version $\psi_{j,{\left\{I_{d}\right\}}_{d=1\ldots p-1}}$ for a finite sample. Similar to the diploid case, this joint SFDS is defined as the number of sites with a derived variant at frequency $f=\frac{j}{pn}$ where the dosage distribution across individuals is $i_{d}=I_{d}/n$. If we condition on a given frequency, we obtain the Site Dosage Spectrum (SDS) *p*({*i*_*d*_}_*d* = 1…*p*−1_|*f*).

**Figure 3:**
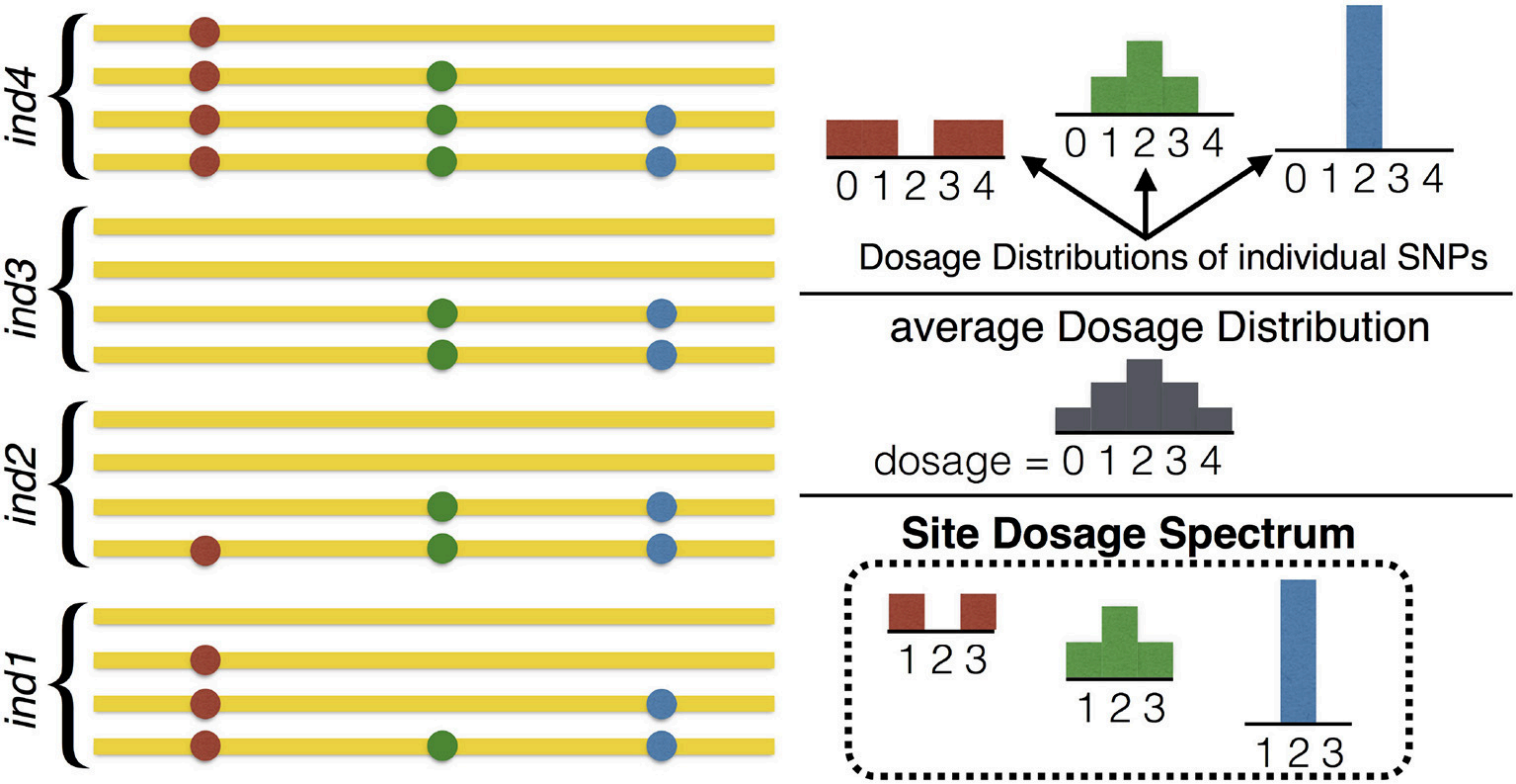
Illustration of the relation between the Dosage Distribution and the Site Dosage Spectrum. On the left, homologous sequences from 4 tetraploid individuals are shown (*n* = 4, *p* = 4), containing 3 SNPs of frequency 50%. On the right, the three DDs (one for each SNP) are shown at the top. The SDS at the bottom is the distribution of these DDs (which in this example is given by the three DDs with probability 1/3 each). Note that the SDS bears no relation with the average DD, which is shown in the middle. In this example, the Site Frequency/Dosage Spectrum would be ψ_8, {1/4, 0, 1/4}_ = 1/3, ψ_8, {1/4, 1/2, 1/4}_ = 1/3, ψ_8, {0, 1, 0}_ = 1/3 and $\psi_{8,\left\{I\right\}}=0$ for other choices of I.

An important and subtle point that should be clear from Figure 3 is that the SDS is the distribution of the DD, and hence it cannot be reliably summarized as a single average DD. Reducing the SFDS for a given frequency to the average DD over all variants of that frequency is the equivalent of summarizing the distribution of heterozygosity in diploids by providing the average heterozygosity only. In fact the SFDS is a full *p*-dimensional spectrum whose components are the frequency (one component) and the heterozygous part of the DD (*p* − 1 components), the latter representing the SDS.

### 2.3. The SFDS of the standard neutral model

The expected value of the SFDS under the standard neutral model is a simple generalization of the diploid frequency-heterozygosity spectrum presented before. In an infinite population and in the absence of double reduction, the Dosage Distribution for a mutation of frequency *f* under Hardy–Weinberg equilibrium is well-known (Haldane, 1930):

(2) id=(pd)fd(1-f)p-d for d=0…p

and the expected value of the neutral SFS has the standard shape

(3) $$\begin{array}{l} E\left[\xi \left(f\right)\right]=\frac{\theta }{f}\;\text{;} \end{array}$$

hence the expected population SFDS is simply

(4) E[ψ(f,{id})]=θf∏d=1p-1δ(id-(pd)fd(1-f)p-d)

where δ(*z*) is the Dirac delta function, which represents a distribution concentrated at *z* = 0.

For finite samples the expected values are slightly more complex. A combinatorial argument similar to the diploid case — based on the ways to assign the *j* mutated alleles across the *pn* homologous chromosomes—provides the following formula for the SDS, i.e., the distribution of the Dosage Distribution ${\left\{I_{d}\right\}}_{d=1\ldots p-1}$ in finite samples of size *n*:

(5) E[p({Id}|j)]=n!I1!I2!…Ip-1!(j-∑d=1p-1dIdp)!(n-jp-(1-1p)(∑d=1p-1dId))!∏d=1p-1(pd)Id(pnj)

where the above expression should be interpreted as 0 if it contains factorials of non-integer numbers. More details can be found in the Appendix.

The SFDS in finite samples can be found combining (5) with the known neutral expected SFS θ/*j*:

(6) $$\begin{array}{l} E\left[\psi_{j,\left\{I_{d}\right\}}\right]=\frac{\theta }{j}\text{E}\left[p\left(\left\{I_{d}\right\}|j\right)\right] \end{array}$$

Note that in finite samples frequency and DD are under the constraint that $j-\overset{p-1}{\underset{d=1}{\sum }}dI_{d}$ should be a multiple of *p*.

## 3. SFS estimators and neutrality tests for large samples

For large samples *n* ≫ 1, the exact shape of the DD and the SDS do often have a negligible impact on tests based on the shape of the SFS and their normalization. In fact, most of these tests place weights on ξ(*f*) that change gradually with the frequency. There are a few exceptions—for instance tests that assign very different weights on singletons, such as Fu and Li's *F* and *D* tests for background selection (Fu and Li, 1993), and the expansion test *R*_2_ (Ramos-Onsins and Rozas, 2002). The shape of Hardy–Weinberg violations affects the SFS on a scale $\Delta f≲\frac{p}{pn}=1/n$. Since most tests weight frequencies in a smooth way over scales of Δ*f* ~ 1/*n* for *n* large enough, the DD can usually be ignored in large samples.

However, unbiased sequence data from a large number of individuals is typically obtained by High-Throughtput Sequencing (HTS) at low to moderate coverage. HTS data at low coverage is usually unbalanced and more prone to be significantly impacted by sequencing errors, thus requiring tailored approaches. Hence in this section we focus on SFS-based estimators of genetic variability and neutrality tests adapted to HTS data.

SNP calling is usually required prior to population genetic analysis. It is even more relevant for HTS data, due to the typical amount of sequencing errors for these technologies. It is key that only methods developed specifically for polyploids (e.g., GATK from Broad Institute) or for pooled data (e.g., Raineri et al., 2012) are used, since the accuracy of SNP calling algorithms depends on the ploidy. Algorithms for diploids are usually unsuitable to analyse data from organisms with higher ploidy.

Allelic dosage estimation could also be performed (e.g., Blischak et al., 2016), but it is unreliable at low coverage and can be challenging even at high coverage. In fact, dosage uncertainties represent one of the biggest hurdles when dealing with polyploid population genetics (Blischak et al., 2016). However, an accurate estimate of allelic dosage for each individual is not needed to estimate genetic diversity at population level. In fact, none of the methods we discuss in this section requires an explicit estimation of dosage. All these methods work directly on short-read data after SNP calling and filtering of unreliable low-frequency variants.

The estimators of variability proposed in this section take read depth explicitly into account and are unbiased at low coverage as well. Hence there is no need to filter regions of low coverage, although excluding regions with read depth lower than the ploidy could increase the accuracy of the results. However, since our estimators do not take sequencing errors into account, we strongly suggest to perform SNP calling prior to analysing variability with them. For such analyses SNPs can be filtered with moderately conservative parameters, e.g., excluding only SNPs with posterior probability >0.95 or equivalently *p*-value >0.05 or PHRED quality score <15.

In this section we consider an experimental setup where every polyploid individual of ploidy *p* in a sample of *n* individuals is sequenced separately with a read depth of *r*_*i*_(*x*) at position *x*, where *i* = 1…*n*. The count of the alternative (derived) alleles within reads from the *i*th individual at position *x* is *c*_*i*_(*x*). If the position *x* has been filtered out during SNP calling, we discard the SNP and consider *c*_*i*_(*x*) = 0 for all individuals.

### 3.1. Estimators of variability

#### 3.1.1. Watterson's estimator

The classical estimator of variability based on the SFS is the Watterson estimator (Watterson, 1975), which is based on the number of segregating sites *S* in a sample of size *n*. Under an infinite sites model and a panmictic stationary and neutral scenario with population size *N*, where mutations are randomly and independently occurring given a mutation rate μ per non-overlapped generation (i.e., a Wright-Fisher model), the expected variability level θ = 2*pN*_*e*_μ can be estimated by:

(7) $$\begin{array}{l} \theta_{W}=\frac{S}{a_{n}}\;\text{,} \end{array}$$

where $a_{n}=\overset{n-1}{\underset{j=1}{\sum }}\frac{1}{j}$. This estimator is based on the expected neutral spectrum of mutations and is sensitive to the presence of an excessive number of singletons (which can be observed, for example, under demographic expansion scenarios (Ramos-Onsins and Rozas, 2002) or in the presence of high rates of artifactual sequencing errors (Achaz, 2008).

A generalization of the Watterson estimator for autopolyploids, in the form of a Maximum Composite Likelihood estimator, has been derived in Equation (34) of Ferretti and Ramos-Onsins (2015). However, this estimator suffers from a strong bias due to sequencing errors. In fact, sequencing errors appear as low frequency variants which increase the estimate of *S*. Two strategies could be applied to reduce this dependence: either *S* should be estimated using only filtered SNPs obtained from SNP calling algorithms, or low frequency variants should be removed with an approach similar to that used in Achaz (2008).

#### 3.1.2. Tajima's estimator of nucleotide diversity

Tajima's estimator (Tajima, 1983) or the pairwise nucleotide difference statistic (Π) is also a relevant estimator of nucleotide diversity and is defined as the average number of differences between sequences. In fact, for each position *i* it estimates the level of heterozygosity in the population [2*f*_*i*_(1 − *f*_*i*_), where *f*_*i*_ is the absolute frequency of a given variant allele at position *i*]. In the infinite-site and stationary neutral model, the expected value of Tajima's estimator (θ_Π_) is equal to that of Watterson's estimator (that is, under the ideal Wright-Fisher scenario E[θ_Π_] = E[θ_*W*_] = θ). Tajima's estimator for a region of size *L* is given by:

(8) $$\begin{array}{l} \theta_{\Pi }=\frac{n}{\left(n-1\right)}\overset{L}{\underset{i=1}{\sum }}2f_{i}\left(1-f_{i}\right). \end{array}$$

Results from Ferretti et al. (2013) can be combined to build an unbiased estimator of pairwise nucleotide diversity for multiple polyploid individuals:

(9) $$\begin{array}{l} {\hat{\theta }}_{\Pi }=\frac{2}{n\left(n-1\right)}\left[\frac{p}{p-1}\overset{n}{\underset{j=1}{\sum }}\pi_{j}+2\overset{n-1}{\underset{j=1}{\sum }}\overset{n}{\underset{k=j+1}{\sum }}\pi_{j,k}\right] \end{array}$$

where π_*j*_ is the average pairwise difference between reads from the *j*th individual, and π_*j,k*_ is the average pairwise difference between pairs of reads from the *j*th and *k*th individual (Ferretti et al., 2013). Both these quantities account naturally for dosage. The factor *p*/(*p* − 1) is the same factor that appears between the estimates of sample and population heterozygosity in the above formula (8) (Nei and Roychoudhury, 1973).

The above estimator weights the information from all individuals equally, irrespectively of their coverage and dosage. It is possible to build less noisy unbiased estimators by considering further assumptions on the variance of the pairwise differences. Given the average coverage per base ${\bar{r}}_{j}$ of the *j*th individual, the variances can be often approximated by inverse powers of this coverage $\text{Var}\left(\pi_{j}\right)\propto 4/{\bar{r}}_{j}+4/p$, $\text{Var}\left(\pi_{j,k}\right)\propto 1/{\bar{r}}_{j}+1/{\bar{r}}_{k}+2/p$ (see Appendix). Hence, an approximate Minimum Variance Unbiased Estimator for the pairwise diversity can be obtained by weighting the terms in the above estimator by their variance:

(10) $$\begin{array}{l} {\hat{\theta }}_{\Pi }=\frac{\sum_{j=1}^{n}\pi_{j}\frac{{\bar{r}}_{j}\left(p-1\right)}{2\left({\bar{r}}_{j}+p\right)}+2\sum_{j=1}^{n-1}\sum_{k=j+1}^{n}\pi_{j,k}{\left(\frac{1}{{\bar{r}}_{j}}+\frac{1}{{\bar{r}}_{k}}+\frac{2}{p}\right)}^{-1}}{\sum_{j=1}^{n}\frac{{\bar{r}}_{j}{\left(p-1\right)}^{2}}{2p\left({\bar{r}}_{j}+p\right)}+2\sum_{j=1}^{n-1}\sum_{k=j+1}^{n}{\left(\frac{1}{{\bar{r}}_{j}}+\frac{1}{{\bar{r}}_{k}}+\frac{2}{p}\right)}^{-1}} \end{array}$$

As both versions of this estimator assign a negligible weight to low frequency alleles, they are much more robust with respect to sequencing errors and uncertainties in SNP calling. Hence in the presence of significant rates of sequencing errors, or other related causes of incorrect base calling, any of these estimators should be preferred to the Watterson estimator discussed above.

### 3.2. Neutrality tests

#### 3.2.1. Tajima's *d*

Tajima's *D* test (Tajima, 1989) was the first neutrality test based on the frequency spectrum and it is still the most popular one. It is based on the difference between the Tajima's estimator θ_Π_ and the Watterson estimator θ_*W*_. As explained above, under the stationary neutral model it is expected that this difference would be zero. However, empirical data violating the theoretical assumptions can result in significant differences. This test can discriminate among some selective and/or demographic processes. The Tajima's *D* statistic is given by:

(11) $$\begin{array}{l} D=\frac{{\hat{\theta }}_{\Pi }-{\hat{\theta }}_{W}}{\sqrt{\text{Var}\left({\hat{\theta }}_{\Pi }-{\hat{\theta }}_{W}\right)}} \end{array}$$

where the denominator is computed under the standard neutral model and is a function of θ and *np*.

For HTS data, the numerator of the test can be simply obtained from the difference of the Tajima's and Watterson's estimators presented above.

Obtaining the exact denominator is computationally tricky. A practical approximation is to use the standard denominator for the test, but replacing the “haploid” sample size *np* by an effective sample size *n*_eff_ defined as the average number of homologous chromosomes that have been actually sequenced at every position, i.e.,

(12) $$\begin{array}{l} n_{\text{eff}}=\frac{1}{L}\overset{L}{\underset{x=1}{\sum }}\overset{n}{\underset{j=1}{\sum }}p\left[1-{\left(1-\frac{1}{p}\right)}^{r_{j}\left(x\right)}\right] \end{array}$$

#### 3.2.2. Fay and wu's *h*

Fay and Wu's *H* test (Fay and Wu, 2000) was designed to detect derived allele frequencies much higher than expected under a neutral scenario. A large number of variants at high frequencies can be a consequence of positive selection, although it could also occur in the presence of signals of population structure (e.g., introgression). The test compares the levels of variability of Tajima's estimator (θ_Π_) vs. another variability estimator—here named θ_*H*_—that weights the number of segregating sites quadratically with the frequency of derived alleles. The normalized version of this test (Zeng et al., 2006) is:

(13) $$\begin{array}{l} H=\frac{{\hat{\theta }}_{\Pi }-{\hat{\theta }}_{H}}{\sqrt{\text{Var}\left({\hat{\theta }}_{\Pi }-{\hat{\theta }}_{H}\right)}} \end{array}$$

For HTS data, we apply the same approach as for Tajima's *D*. The only difference is that we use the alternative definition of the numerator 2(θ_Π_ − θ_*L*_) where θ_*L*_ is the Zeng's estimator, which is linear in the derived frequency (Zeng et al., 2006). An unbiased version of θ_*L*_ for HTS data is

(14) $${\hat{\theta }}_{L}=\sum_{x=1}^{L}\frac{\sum_{j=1}^{n}c_{j}(x)}{N_{L}(x)\sum_{j=1}^{n}r_{j}(x)}$$

where the normalization factor

(15) $$N_{L}=\sum_{k=1}^{pn-1}\frac{1}{k}\sum_{k_{1}=0}^{p}\ldots \sum_{k_{n}=0}^{p}\delta_{k,k_{1}+\ldots +k_{n}}\frac{\prod_{i=1}^{n}\left(\begin{array}{c} p\\ k_{i} \end{array}\right)}{\left(\begin{array}{c} pn\\ k \end{array}\right)}\left[1-\prod_{i=1}^{n}{\left(\frac{k_{i}}{p}\right)}^{r_{i}(x)}\right]$$

is the probability that a segregating site is not interpreted as a fixed derived variant based on the reads. Note that δ_*i,j*_ is the Kronecker delta which is 1 if *i* = *j* and 0 otherwise.

An approximate version of the denominator of the test can be derived inserting *n*_eff_ in the standard denominator, as described above for Tajima's *D*.

## 4. Small samples and hardy–weinberg violations in the SDS

For small autopolyploid samples, deviations from the neutral SFS cannot be clearly discriminated from violations of Hardy–Weinberg. In fact, in the smallest possible sample of a single individual, the Dosage Distribution coincides with the SFS! More precisely, the SFS for a single individual corresponds to the heterozygous components of the Dosage Distribution averaged across sites. Hence, the features of the DD have a huge impact on the SFS.

This impact is two-fold. On a practical side, if it is not possible to estimate allelic dosage with sufficient accuracy, then uncertainties in individual dosage result in large uncertainties in the determination of allele frequencies, and therefore of the SFS. However in principle, even if dosage could be accurately inferred, the shape of the SFS for a few individuals would still be largely determined by the effect on the DD of the deviations from Hardy–Weinberg equilibrium. We will discuss such deviations in this section.

For diploid organisms there is only one possible direction for Hardy–Weinberg violation, i.e., excess or deficit of heterozygotes. However, in autopolyploids, many different deviations from Hardy–Weinberg equilibria are possible, resulting in different deviations from the neutral SFS. In fact, in this section we present four examples of possible mechanisms of violation of Hardy–Weinberg equilibrium which correspond to four different directions in the space of expected DDs. These examples are (i) inbreeding; (ii) inbreeding with mixed disomic/polysomic inheritance; (iii) heterozygote advantage; (iv) selection against recessive mutations. In tetraploids, combinations of these mechanisms span the whole space of all possible deviations from Hardy–Weinberg.

**Figure 4:**
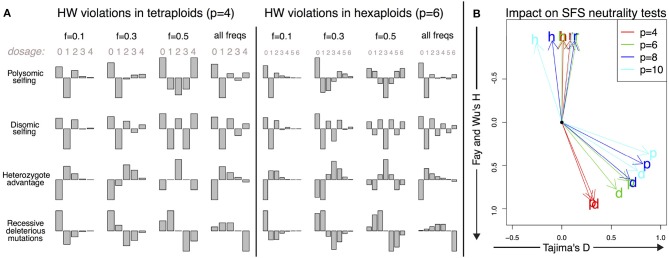
Deviations from the Hardy–Weinberg equilibrium and their impact on the DD. **(A)** Shape of the small deviations $\Delta I_{k}$ of the DD from the Hardy–Weinberg equilibrium for both tetraploid and hexaploid individuals in four different scenarios: polysomic selfing (p); disomic selfing (d); heterozygote advantage (h); recessive deleterious mutations (r). We show the deviations for mutations of given frequency (0.1, 0.3, and 0.5) together with the expected violations for random neutral mutations of arbitrary frequency (i.e., distributed as θ/*f*). The absolute amplitude of the deviations is arbitrarily chosen for each plot; its actual value will depend on parameters such as selfing rates and selection coefficients. **(B)** Impact of the deviations on SFS-based neutrality tests for a single individual. The overall impact is proportional to the amplitude of the deviations; here we show only the directions of apparent violation of neutrality along the space of two SFS-based tests (Tajima's *D* and Fay and Wu's *H*). The expected deviations from neutrality are shown for the same four scenarios as in **A** (p, d, h, and r) and for tetraploid, hexaploid, octoploid and decaploid organisms. The black dot corresponds to the neutral values *D* = 0 and *H* = 0.

The shapes of the deviations of the expected DD from a Hardy–Weinberg equilibrium are shown for these mechanisms in Figure 4, both in tetraploids and hexaploids. The corresponding directions of the deviations of SFS-based tests from their null values are shown in the same figure for Tajima's *D* and Fay and Wu's *H* for a range of ploidy from 4 (tetraploids) to 10 (decaploids).

### 4.1. Inbreeding

Inbreeding is a well-known cause of violation of Hardy–Weinberg. Both in diploids and in polyploids, selfing and other mechanisms such as subpopulation structure cause a lack of heterozygotes, as discussed in relation to the Wahlund effect (Rosenberg and Calabrese, 2004).

As an example of its consequences on the DD, we can model a small rate of selfing in a population with polysomic inheritance by assuming an equilibrium in the DD given the frequency of the variant, with an approach similar to the one used in De Silva et al. (2005):

(16) $$\begin{array}{l} I_{k}^{\text{eq}}=\overset{p}{\underset{k^{\prime }=0}{\sum }}\overset{p}{\underset{k^{\prime \prime }=0}{\sum }}I_{k^{\prime }}^{\text{eq}}I_{k^{\prime \prime }}^{\text{eq}}\overset{p}{\underset{a=0}{\sum }}\text{Hyp}\left(a|k^{\prime },p/2,p\right)\text{Hyp}\left(k-a|k^{\prime \prime },p/2,p\right) \end{array}$$

where Hyp(·) is the hypergeometric distribution that corresponds to the sampling of chromosomes in gametes. Note that all the Hardy–Weinberg equilibrium distributions Ikeq=(pk)fk(1-f)p-k discussed before are solutions of the equation above (Here and in the rest of this section, we ignore the possibility of double reduction, since it requires a separate modeling of its impact on allele frequencies; Butruille and Boiteux, 2000).

Then we can perturb the equilibrium by occasional selfing events with a small probability *p*_*s*_, obtaining:

(17) $$\begin{array}{l} \Delta I_{k}=-p_{s}I_{k}^{\text{eq}}+p_{s}\overset{p}{\underset{k^{\prime }=0}{\sum }}I_{k^{\prime }}^{\text{eq}}\overset{p}{\underset{a=0}{\sum }}\text{Hyp}\left(a|k^{\prime },p/2,p\right)\text{Hyp}\left(k-a|k^{\prime },p/2,p\right) \end{array}$$

The shape of this violation of Hardy–Weinberg is shown in Figure 4. As expected, it results in an excess of homozygotes in the population. For a single individual, it has a positive impact on both Fay and Wu's *H* and Tajima's *D*. For tetraploids, the deviations from the null value are more apparent in *H*, while in organisms with ploidy higher than 6, violations tend to be larger in *D*.

### 4.2. Intermediate disomic/polysomic inheritance

Not only the rates of selfing/outcrossing, but also the mode of inheritance could impact on the violation of Hardy–Weinberg. Mixed disomic/polysomic inheritance is an example of an alternative inheritance mode that appears to be less rare than expected (Meirmans and Van Tienderen, 2013).

Without inbreeding, partial disomic inheritance alone does not lead to violations of the Hardy–Weinberg equilibrium. Hence to study deviations from Hardy–Weinberg we model mixed disomic/polysomic inheritance but with a small selfing rate *p*_*s*_, similar to the case above. We denote the probability of disomic and polysomic inheritance by *p*_2_ and 1 − *p*_2_ respectively. For small selfing rate, it is easy to argue that the violations would be a combination of purely disomic and purely polysomic violations with weights *p*_2_ and 1 − *p*_2_ respectively, i.e.,

(18) $$\begin{array}{l} \Delta I_{k}=\left(1-p_{2}\right)\Delta I_{k}^{polysomic}+p_{2}\Delta I_{k}^{disomic} \end{array}$$

assuming that *p*_*s*_ ≪ 1.

Purely disomic violations would satisfy similar equations as the purely polysomic ones in the previous section, although with slightly different inheritance terms. Similar to what happens in diploid organisms, sampling of the new generation occurs separately for each heterozygous pair of disomically homologous chromosomes:

(19) $$\Delta I_{k}=-p_{s}I_{k}^{\text{eq}}+p_{s}\sum_{k^{\prime }=0}^{p}I_{k^{\prime }}^{\text{eq}}\sum_{h=0}^{p/2}\frac{2^{h}\left(\begin{array}{c} p/2\\ h;\frac{k^{\prime }-h}{2};\frac{p-k^{\prime }-h}{2} \end{array}\right)}{\left(\begin{array}{c} p\\ k^{\prime } \end{array}\right)}\left(\begin{array}{c} h\\ \frac{k-k^{\prime }+h}{2} \end{array}\right)2^{-h}$$

The corresponding shape of Hardy–Weinberg violations shown in Figure 4 is similar to the one of selfing in polysomic organisms, but with an excess of homozygous pairs of disomically homologous chromosomes that translates into an excess in the components of even dosage in the spectrum. The impact on Fay and Wu's *H* and Tajima's *D* is similar to that of purely polysomic inheritance.

### 4.3. Heterozygote advantage

Heterozygote advantage, or overdominance, is a form of “hybrid vigor” where individuals heterozygous for the locus considered acquire a higher fitness than those provided by the two homozygous genotypes. For simplicity, we can assume the two differences in fitness to be the same. Unsurprisingly, this effect tends to increase the amount of intermediate-frequency alleles and heterozygotes (Kaplan et al., 1988).

Modeling selection dependent on the allelic dosage can be done via an approach similar to the one employed above, but is trickier. Selection is not a one-off or rare event but perturbs permanently the equilibrium $I_{k}^{\text{eq}}$, hence a self-consistent version of the perturbative equations should be employed. Assigning a fitness ϕ_*k*_ = 1 + *s*_*k*_ to each allelic dosage, we obtain the equilibrium condition

(20) $$\begin{array}{l} I_{k}^{\text{eq}}=\overset{p}{\underset{k^{\prime }=0}{\sum }}\overset{p}{\underset{k^{\prime \prime }=0}{\sum }}\frac{I_{k^{\prime }}^{\text{eq}}\phi_{k^{\prime }}I_{k^{\prime \prime }}^{\text{eq}}\phi_{k^{\prime \prime }}}{{\left(\sum_{l=0}^{p}I_{l}^{\text{eq}}\phi_{l}\right)}^{2}}\overset{p}{\underset{a=0}{\sum }}\text{Hyp}\left(a|k^{\prime },p/2,p\right)\text{Hyp}\left(k-a|k^{\prime \prime },p/2,p\right) \end{array}$$

We can then perturb at linear order in *s*_*k*_ and compute $\Delta I_{k}=I_{k}^{\text{eq}}-I_{k}^{0}$, with $I_{k}^{0}$ being a solution of Equation (16). After using the fact that $\overset{p}{\underset{k=0}{\sum }}I_{k}^{0}=1$, we obtain the linear system

(21) $$\begin{array}{l} \Delta I_{k}=2\overset{p}{\underset{k^{\prime }=0}{\sum }}\overset{p}{\underset{k^{\prime \prime }=0}{\sum }}I_{k^{\prime }}^{0}\left(I_{k^{\prime \prime }}^{0}s_{k^{\prime \prime }}+\Delta I_{k^{\prime \prime }}\right)\times \\ \overset{p}{\underset{a=0}{\sum }}\text{Hyp}\left(a|k^{\prime },p/2,p\right)\text{Hyp}\left(k-a|k^{\prime \prime },p/2,p\right)\\ -2I_{k}^{0}\overset{p}{\underset{l=0}{\sum }}\left(I_{l}^{0}s_{l}+\Delta I_{l}\right) \end{array}$$

This equation describes how perturbations to the neutral equilibrium driven by weak selection increase, which is a good proxy for the shape of Hardy–Weinberg violations in the DD.

An example of a fitness assignment that leads to heterozygote advantage is *s*_*k*_ = *s* for *k* = 1…*p* − 1 but *s*_0_ = 0, *s*_*p*_ = 0. This gives a constant fitness advantage to all heterozygotes, independently on their dosage.

We report the Hardy–Weinberg violations for this example in Figure 4. As expected, heterozygote advantage increases the number of alleles at all frequencies while reducing homozygotes. Surprisingly enough, despite the intuition that the effect would be to increase Tajima's *D* due to the excess of intermediate-frequency variants, the final spectrum impacts negatively on Fay and Wu's *H* and only weakly on Tajima's *D*, as shown in Figure 4.

### 4.4. Recessive deleterious mutations

It is possible to use the same approach as in the previous subsection to deal with selection against derived homozygotes. If the mutation is deleterious but recessive, there will be a fitness gap between the homozygotes for the derived allele, which would show the phenotypic effects of the mutation, and all other genotypes, that would not. This is another classical cause of violation of Hardy–Weinberg equilibrium, although in practice it is difficult to detect since the mutations involved tend to be at low frequency and therefore the lack of derived homozygotes could be attributed to the Hardy–Weinberg equilibrium itself.

The fitness assignment for a recessive deleterious allele is *s*_*p*_ = −*s* but *s*_*k*_ = 0 for *k* = 0…*p* − 1. This describes a selection pressure against derived homozygotes only.

The shape of the Hardy–Weinberg violations in this case shows the expected reduction in derived homozygotes and an excess in intermediate-dosage heterozygotes. This causes a reduction in Fay and Wu's *H*, as shown in Figure 4. Ironically, negative values of Fay and Wu's *H* are also one of the typical signatures of selection and genetic hitchhiking.

## 5. Discussion

In order to advance our understanding of the evolutionary processes affecting the genome of polyploid species, an important step is to gain a deeper knowledge of the way these processes modulate the fate of genetic variants, and consequently the levels and patterns of genetic variability. Two of the main descriptive statistics used in population genetics to summarize genetic variability are the SFS and the heterozygosity (*h*), which contain information on the global and internal allelic spectra, respectively. The expected patterns of these statistics have not been studied in detail for polyploids; that is especially true for many conditions commonly found in empirical studies of autopolyploid species, for instance small sample sizes and violations of the Hardy–Weinberg equilibrium such as inbreeding. In addition, understanding the expected patterns in commonly used statistics such as Tajima's *D* or Fay and Wu's *H* tests is of great relevance for the correct interpretation of the evolutionary processes occurring in autopolyploid populations. Typical patterns there could well be different from the expected patterns in diploid populations, simply because genetic and evolutionary processes have different peculiarities in the two cases.

Studies focused on the analysis of nucleotide variability in polyploid species present special difficulties in comparison to diploid species, as is extensively reviewed in Dufresne et al. (2014). These difficulties have been partially the reason for a relatively scarce number of publications on HTS analysis of genomic variability among wild autopolyploid populations. Nevertheless polyploid plant species in particular are of great interest, given their high economic and strategic impact. In the last years there has been a proliferation of studies on related model species such as *Arabidopsis* (e.g., Hollister et al., 2012; Arnold et al., 2015), other relatively simple species (e.g., Cornille et al., 2016; Kasianov et al., 2017), but also economically important species with more complex genetics (e.g., Raman et al., 2014; Rocher et al., 2015; Kamneva et al., 2017; Krasileva et al., 2017). Although the number of relevant datasets deposited in sequence databases is constantly growing, their adequate analysis will require the further development of specific statistical tools, especially to infer sequence variability and population genomics.

In this manuscript we outlined the rich structure of frequency spectra in autopolyploids. The combination of global and internal spectra—i.e., mutation frequency in the population for the SFS, and allelic dosage in individuals for the SDS—contributes to the complexity of the polyploid SFDS.

The intricacy of the SFS structure and the challenges posed by its correct inference are possibly the reasons why this summary statistic has been given scant attention in polyploids so far (Dufresne et al., 2014; Meirmans et al., 2018), despite the fact that it represents one of the classical statistics in population genetics (Nielsen, 2005; Casillas and Barbadilla, 2017).

In this paper we also discussed some of the challenges related to the analysis of autopolyploid data generated by HTS technologies. However, our discussion is restricted to the simplified case of Hardy–Weinberg equilibrium, which is likely to be violated in many real populations of autopolyploid plants e.g., because of selfing. Even for purely outcrossing autopolyploid organisms, violations of Hardy–Weinberg could be caused by widespread mechanisms such as a large number of recessive deleterious alleles. Similarly, the interplay between the SFS and the Dosage Distribution has been discussed here only in the simplified case of small perturbations of Hardy–Weinberg equilibrium in a single individual. These assumptions allow us to present for the first time a systematic picture of the issues; on the other hand, more work is required to build a theoretical understanding of the SFDS and of SFS-based inference in polyploids, especially for small samples.

One of the most important consequences of the present work is the different interpretation of the neutrality test under deviations from a neutral panmictic model in Hardy–Weinberg equilibrium (Figure 4). For a low number of samples, the SFS tends to be dominated by the SDS. Deviations from Hardy–Weinberg equilibrium within each individual distort the full SFS and result in values of neutrality tests that are different from those expected in diploid populations undergoing the same processes. For instance, heterozygote advantage in a small sample of diploid individuals is expected to result in an increase of heterozygotes and therefore a deviation of the Tajima's *D* test toward positive values. On the other hand, in a single autopolyploid individual with the same number of homologous chromosomes, this effect would be close to zero or negative. The reason is two-fold: homozygote alleles would not be classified as polymorphisms and therefore would not be included in the spectrum, while the impact of heterozygote advantage on dosage itself is complex. Generally speaking, the impact of Hardy–Weinberg violations on allelic dosage tends to affect deeply the SFS of the global sample when the sample size is small, complicating the interpretation of the results of neutrality tests. Note that the Hardy–Weinberg equilibrium is not reached in a single generation for autopolyploid species, leaving a longer signal in the genome patterns in relation to diploid species.

The role of allelic dosage uncertainties should be emphasized once more. Despite being challenging, the inference of individual genotypes (i.e., allelic dosage) by likelihood estimation can be obtained from HTS datasets using several algorithms. Recently, Maruki and Lynch (2017) developed a genotype calling algorithm that has proven useful for population genetic analysis. Nevertheless, accurate inference can only be obtained with high read depths and high cost, which usually implies the analysis of just a few individuals. Even in such a case, as shown in this paper, the inference of genotype likelihoods could be hindered by conservative assumptions on the Hardy–Weinberg patterns of the DD, which can generate systematic biases especially in relation to low frequency variants. Focusing on the analysis of variability, the real genotype of each individual is not as important as the pattern of the whole SFS, considering the uncertainties produced by deviations from Hardy–Weinberg equilibrium and other random processes. That is the reason why the equations presented here make performing genotype inference for each autopolyploid individual unnecessary.

Another reason why allelic dosage uncertainty is not a limitation for SFS inference can be illustrated by the following general argument. By definition, the frequency of an allele is the sum of its allelic dosages across individuals divided by the total number of homologous chromosomes in the sample, i.e., *np*. This implies a relation between frequencies and their uncertainties: more precisely, by classical probability arguments, the standard deviation of the frequency is the quadratic mean of the standard deviation of the allelic dosage divided by $p\sqrt{n}$. Hence, no matter how large is the allelic dosage uncertainty for each individual, the accuracy in the reconstruction of the frequency is always good for samples of large enough size. In fact, the maximum standard deviation of allelic dosage is *p*/2, i.e., the uncertainty in frequency is at most $\frac{1}{2\sqrt{n}}$. This means that 25 individuals are sufficient to estimate allele frequencies with an uncertainty of about 0.1, even in the worst-case estimate of allelic dosage uncertainties.

How large the actual sample should be depends on the actual uncertainties in dosage and the evolutionary dynamics of the population. The typical uncertainties in dosage inference from HTS are expected to be around $p/\sqrt{\bar{r}}$ where $\bar{r}$ is the average read depth per individual, hence they decrease with the sequencing depth of the experiment. However, if the dynamics is driven by rare variants, a larger number of individuals is needed to obtain an accurate estimate of their frequency, since the unavoidable variance in frequency due to the sampling process of individuals from the whole population is between $\frac{f\left(1-f\right)}{pn}$ (under Hardy–Weinberg equilibrium) and $\frac{f\left(1-f\right)}{n}$ (if the Hardy–Weinberg conditions are strongly violated).

At present, the complexity of most analyses implies that good-quality population genetic data of samples of multiple autopolyploid organisms from the same natural population are hard to obtain. Most of the efforts so far were focused on the relation between different populations (Meirmans and Hedrick, 2011) and the comparison between different levels of ploidy, which require the sequencing of single samples from multiple populations. On a broader evolutionary scale, polyploidization during speciation and its evolutionary consequences were also studied in several biological systems (Parisod et al., 2010; Barker et al., 2016). However, there is a general lack of good datasets, and theoretical approaches to understand the microevolutionary picture are lagging behind (Dufresne et al., 2014; Meirmans et al., 2018), with the possible exception of linkage and QTL mapping. We hope that this paper will raise some awareness of the issues involved and clarify the relation between important quantities such as the frequency spectrum, the heterozygosity and the distribution of allelic dosage.

In conclusion, considering spectra of allelic dosage such as the SDS is of fundamental importance for the study of the evolutionary processes in autopolyploids. These internal spectra have a large impact on the global SFS for small sample sizes (for large sample size, the SFS can be reliably inferred and should not be strongly affected by Hardy–Weinberg violations). In this framework, we have proposed a set of estimators of variability and neutrality tests for autopolyploid HTS samples, based on well-known tests such as Tajima's *D* and Fay and Wu's *H*. Additionally, we have shown how different deviations from Hardy–Weinberg equilibrium and other uncertainties are reflected in the dosage distribution at the level of single individuals. In general, we bring attention to the importance of the study of the joint SFDS in polyploid species in order to correctly interpret the patterns of population variability.

## Author contributions

LF and SR-O conceived the paper. LF and PR developed the theory. LF implemented it. LF, PR, and SR-O wrote the paper.

### Conflict of interest statement

The authors declare that the research was conducted in the absence of any commercial or financial relationships that could be construed as a potential conflict of interest.
