# Supplementary material for: The Site Frequency/Dosage Spectrum of Autopolyploid Populations
Source: Front Genet. 2018 Oct 23;9:480. doi: 10.3389/fgene.2018.00480 (PMC6207136; doi:10.3389/fgene.2018.00480)
Supplement: Supplementary file 1 [file Data_Sheet_1.pdf]

APPENDIX

Derivation of the expected SDS under Hardy-Weinberg equilibrium

The SDS for finite samples under Hardy-Weinberg equilibrium can be derived by combinatorial arguments. More precisely, for biallelic variants the Hardy-Weinberg equilibrium is equivalent to a random sampling process whereby the p alleles for each individual are independently sampled from an infinite pool containing only two types of alleles. If we condition on the number of alleles of each type sampled in the whole sample (j and $pn-j$ respectively) then the probability of a given configuration of DD $\mathcal{I}_d$ can be found as the ratio of the number of choices of j alleles that return the DD $\mathcal{I}_d$ versus the number of all possible choices of j alleles of the first type out of pn. The latter contribution is $\binom{pn}{j}$, while the former contribution can be found by multiplying the multinomial coefficient describing the number of ways to distribute the m individuals among all dosage classes according to the DD $\mathcal{I}_d$, i.e.

$$\frac{n!}{\mathcal{I}_1!\mathcal{I}_2!\ldots\mathcal{I}_{p-1}!\left(\frac{j-\sum_{d=1}^{p-1}d\mathcal{I}_d}{p}\right)!\left(n-\frac{j}{p}-\left(1-\frac{1}{p}\right)\left(\sum_{d=1}^{p-1}d\mathcal{I}_d\right)\right)!},$$

by the binomial coefficients describing the number of ways to distribute the d_x alleles between the p homologous chromosomes of the xth individual,

$$\prod_{x=1}^{n}\binom{p}{d_x}=\prod_{d=0}^{p}\binom{p}{d}^{\mathcal{I}_d}=\prod_{d=1}^{p-1}\binom{p}{d}^{\mathcal{I}_d}.$$

Putting together the two contributions, we find that their ratio corresponds to equation (5).

Derivation of the HTS estimators of variability

Derivation of Tajima's estimators for autopolyploids

A polyploid individual from a population in Hardy-Weinberg equilibrium behaves similarly to a pooled sample of p haploid individuals. Both $\frac{p}{p-1}\pi_j$ (where π_j is the average pairwise difference between reads from the jth individual) and $\pi_{j,k}$ (the average pairwise difference between pairs of reads from the jth and kth individual) are unbiased estimators of θ under Hardy-Weinberg equilibrium, as implied by the results on unbiased estimators for pooled samples from Ferretti et al. (2013). Weighting all individuals and ordered pairs of individuals equally, we obtain the estimator (9). However, it is possible to improve this estimator by considering the variance of each estimator. Approximating the estimators as independent, the Minimum Variance Unbiased Estimator corresponds to the linear combination

$$\hat{\theta}_\Pi=\frac{\frac{p-1}{p}\sum_{j=1}^{n}\pi_j/\mathrm{Var}[\pi_j]+\sum_{j=1}^{n-1}\sum_{k=j+1}^{n}\pi_{j,k}/\mathrm{Var}[\pi_{j,k}]}{\left(\frac{p-1}{p}\right)^2\sum_{j=1}^{n}1/\mathrm{Var}[\pi_j]+\sum_{j=1}^{n-1}\sum_{k=j+1}^{n}1/\mathrm{Var}[\pi_{j,k}]}$$

Exact formulae for the variance of π_j are available in Ferretti et al. (2013). However, for simplicity, we approximate the variances by the delta method. In this approximation, $\mathrm{Var}[\pi_j]\approx 4(1-2f_j)^2\mathrm{Var}[f_j]$ and $\mathrm{Var}[\pi_{j,k}]\approx(1-2f_j)^2\mathrm{Var}[f_k]+(1-2f_k)^2\mathrm{Var}[f_j]$ where f_j denotes here the fraction of reads containing the derived allele. The variance of the number of reads is the result of a double sampling process (sampling of homologous chromosomes from the population, then sampling of reads from homologous

chromosomes). This double binomial sampling gives $\mathrm{Var}[f_k] \approx \left(\frac{1}{r}+\frac{1}{p}\right) f_k(1-f_k)$ where each term of the sum corresponds to a part of the sampling process. Assuming all frequencies to be similar, we obtain the simple results $\mathrm{Var}(\pi_j) \propto 4(1/r_j + 1/p)$, $\mathrm{Var}(\pi_{j,k}) \propto 1/r_j + 1/r_k + 2/p$, where the common multiplicative factor is irrelevant. Inserting such variances in the above equation, we obtain the estimator (10).

Derivation of the formula for the effective sample size

The number of homologous chromosomes sampled is the sum of indicator variables (one for each chromosome) of value 1 if the chromosome has been sampled and 0 otherwise. By the linearity of expectations, the expected number of homologous chromosomes sampled with $r_j(x)$ sequencing reads (coming from individual j, and covering position x in the genome) corresponds to the number of homologous chromosomes p for that individual multiplied by the probability of sampling a given chromosome. The complementary probability of not sampling a given chromosome is $(1-1/p)^{r_j(x)}$. Hence, averaging over all positions, we obtain equation (12).

Derivation of Zeng's estimator for autopolyploids

For each position x, the expectation of $c_j(x)$ conditional on the dosage d_j of the jth individual is $r_j(x)\frac{d_j}{p}$ from binomial sampling. The expectation of the dosage is $\sum_{d=1}^{p} d\frac{\theta}{d} = \theta p$, hence the expectation of $\sum_{j=1}^{n} c_j(x)$ would be $\theta \sum_{j=1}^{n} r_j(x)$. However, note that the case where $c_j(x) = r_j(x)$ for every individual j would not be considered as a polymorphism in the statistic, hence we have to remove the contribution of such cases. The normalisation factor is precisely the complementary of the probability of these cases. The normalisation factor can be obtained from the same approach as in Ferretti and Ramos-Onsins (2015); in fact, it is just a simplification of the normalisation of equation 34 therein.

Derivation of the Hardy-Weinberg violations in the DD

Derivation of the DD with polysomic inheritance and selfing

The equilibrium condition can be derived by requiring that the dosage $\mathcal{I}_k^{\mathrm{eq}}$ should be equal to the same dosage after a generation of crossing with polysomic inheritance. The dosage of each parent is chosen according to the same distribution $\mathcal{I}_k^{\mathrm{eq}}$, then half of the chromosomes are sampled from each of the two parents (resulting in a hypergeometric distribution $\mathrm{Hyp}(a|k', p/2, p)$ for the allele count a) and combined to form the dosage of the offspring. This leads to equation (16). The resulting equation (16) is quadratic in $\mathcal{I}_k^{\mathrm{eq}}$ and is solved by all combinations of Hardy-Weinberg distributions $\mathcal{I}_k^{\mathrm{eq}} = \binom{p}{k} f^k (1-f)^{p-k}$.

A single selfing event would remove an individual with DD $\mathcal{I}_k^{\mathrm{eq}}$ and replace it with an offspring with $p/2$ and $p/2$ chromosomes sampled from the same parent. By multiplying this by the (small) probability of selfing p_s, we find equation (17). Since p_s is an overall factor, the shape of the violations of Hardy-Weinberg in the DD depends only on the initial equilibrium DD $\mathcal{I}_k^{\mathrm{eq}}$.

Derivation of the DD with disomic inheritance and selfing

This derivation is similar to the previous one, except for the sampling term from a single parent during selfing. Such term is the product of the probability that the parent has h heterozygote genotypes among the disomically inherited pairs of chromosomes — which corresponds to $\frac{\binom{p/2}{h;\frac{k'-h}{2};\frac{p-k'-h}{2}} \cdot 2^h}{\binom{p}{k'}}$ by standard combinatorial arguments on the way to distribute the derived alleles among the $p/2$ pairs and then among

the h heterozygotes — and the binomial distribution of the number of heterozygotes that survive the selfing process — which corresponds to $\binom{h}{\frac{k-k'+h}{2}}2^{-h}$.

For mixed disomic/polysomic inheritance, as long as the selfing probabilities are small, the joint probability of polysomic and disomic selfing is negligible, then the two violations do not interfere and the overall violation is a linear combination of the disomic and polysomic ones.

Derivation of the DD with polysomic inheritance and selection

The derivation for the equilibrium condition (20) is similar to the case without selection; however, in this case selection on dosage changes the choice of parents. Individuals are chosen as parents with probability proportional to their fitness, and hence the dosage distribution of parents is weighted by the fitness ϕ_d of each dosage class: $\frac{\mathcal{I}_d^{\mathrm{eq}}\phi_d}{\sum_{l=0}^{p}\mathcal{I}_l^{\mathrm{eq}}\phi_l}$. The rest of the derivation proceeds as in the case without selection.

For the purpose of this paper we made no attempt at solving the non-linear equation (20). Instead, we expanded the equation at first order for small selection coefficients s_k and small deviations $\mathcal{I}_k^{\mathrm{eq}} - \mathcal{I}_k^0$ from the Hardy-Weinberg equilibrium, denoted by $\mathcal{I}_k^0$. The resulting equation (21) is a linear non-homogeneous system for $\Delta\mathcal{I}_k$. Through numerical tests for every selective pressure and ploidy analysed in this paper, we found out that the homogeneous system always contains non-trivial null eigenvalues. Hence the equilibrium under selection cannot be solved perturbatively, as small perturbations corresponding to such null eigenvalues are actually unstable and tend to grow in time. However, the leading term driving this growth provides a good illustration of the typical shape of the corresponding Hardy-Weinberg violations. To find it, we add a small term $-\varepsilon\Delta\mathcal{I}_k$ (with $\varepsilon \ll 1$) to the r.h.s. of equation (21), which allows to solve the inhomogeneous linear system. The resulting stationary solution diverges as $1/\varepsilon$. We extract this divergent contribution; from the theory of matrix differential equations, it corresponds to the leading term by which Hardy-Weinberg violations increase.
